# Indoor versus outdoor scene recognition for navigation of a micro aerial vehicle using spatial color gist wavelet descriptors

**DOI:** 10.1186/s42492-019-0030-9

**Published:** 2019-11-26

**Authors:** Anitha Ganesan, Anbarasu Balasubramanian

**Affiliations:** Department of Aerospace Engineering, Madras Institute of Technology, Anna University, Chennai, 600 044 India

**Keywords:** Micro aerial vehicle, Scene recognition, Navigation, Visual descriptors, Support vector machine

## Abstract

In the context of improved navigation for micro aerial vehicles, a new scene recognition visual descriptor, called spatial color gist wavelet descriptor (SCGWD), is proposed. SCGWD was developed by combining proposed Ohta color-GIST wavelet descriptors with census transform histogram (CENTRIST) spatial pyramid representation descriptors for categorizing indoor versus outdoor scenes. A binary and multiclass support vector machine (SVM) classifier with linear and non-linear kernels was used to classify indoor versus outdoor scenes and indoor scenes, respectively. In this paper, we have also discussed the feature extraction methodology of several, state-of-the-art visual descriptors, and four proposed visual descriptors (Ohta color-GIST descriptors, Ohta color-GIST wavelet descriptors, enhanced Ohta color histogram descriptors, and SCGWDs), in terms of experimental perspectives. The proposed enhanced Ohta color histogram descriptors, Ohta color-GIST descriptors, Ohta color-GIST wavelet descriptors, SCGWD, and state-of-the-art visual descriptors were evaluated, using the Indian Institute of Technology Madras Scene Classification Image Database two, an Indoor-Outdoor Dataset, and the Massachusetts Institute of Technology indoor scene classification dataset [(MIT)-67]. Experimental results showed that the indoor versus outdoor scene recognition algorithm, employing SVM with SCGWDs, produced the highest classification rates (CRs)—95.48% and 99.82% using radial basis function kernel (RBF) kernel and 95.29% and 99.45% using linear kernel for the IITM SCID2 and Indoor-Outdoor datasets, respectively. The lowest CRs—2.08% and 4.92%, respectively—were obtained when RBF and linear kernels were used with the MIT-67 dataset. In addition, higher CRs, precision, recall, and area under the receiver operating characteristic curve values were obtained for the proposed SCGWDs, in comparison with state-of-the-art visual descriptors.

## Introduction

**Fig. 1:**
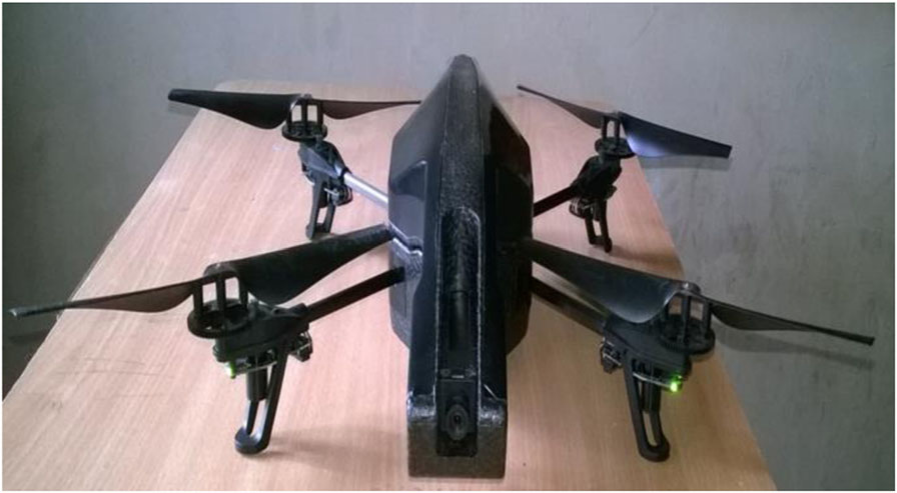
Parrot augmented reality drone2 quadrotor

Classification of a scene as being indoors or outdoors is a challenging task in the navigation of a micro aerial vehicle (MAV). Outdoor scenes, due to different weather conditions and the wide variety of objects involved, as well as their unstructured nature, are much harder to recognize than indoor scenes. Better indoor versus outdoor scene categorization will help MAV navigation where global positioning systems (GPS) signals are not available. Blockage or interruption of GPS signals by dense and tall buildings, by trees and inside buildings occurs in both the indoor and outdoor environment, and to overcome this limitation, the MAV (Fig. 1) needs to have the ability to recognize the difference. A scene classification method has been proposed for indoor versus outdoor scene classification, using a probabilistic neural network that extracts color features, texture features and shape features [1] from the indoor and outdoor images.

Others have considered this issue. Payne and Singh [2] proposed a method based on edge straightness information, extracted from indoor and outdoor images, for efficient indoor and outdoor image classification. A circular thresholding method [3], based on Otsu’s algorithm, used color features, texture features and Otsu features, for indoor versus outdoor image classification. Semantic, high-level, scene image properties [4] can be identified in low level features extracted from the sub blocks of an image, and Quattoni and Torralba [5] used local and global spatial properties of a scene for indoor scene recognition. Videos of indoor environments (corridors, staircases, and rooms), captured by the forward-facing camera of the MAV (Fig. 1) were transmitted, with an image resolution of 1280 × 720 pixels, via a Wi-Fi ad-hoc connection between an augmented reality (AR) drone and a ground station. Another indoor scene classification method [6] has been proposed that combines orientational pyramid matching features with spatial pyramid matching (SPM) features, using 3 dimensional orientational features to discriminate between confusing indoor scene categories.

Nearest neighbor-based metric functions [7] and Bag-of-visual word schemes have been used to recognize indoor scenes, while local image regions have been represented by contextual visual words [8] for scene categorization. In another study, a Scene Understanding (SUN) database [9] was proposed for large-scale scene recognition, using 130,519 images from 899 scene categories.

Efficient scene recognition methods [10–12] have been proposed by researchers across the world. Recently, region-based contextual visual information, integrated with the Bag-of-visual words approach and contextual visual word-based image representation [13] of the scene, was proposed for efficient scene categorization. In another study, a query binary image retrieval method [14] was proposed, using deep belief networks and a Softmax classifier, while neural network classifiers [15] have been used as a tool to categorize remotely sensed images.

A divisive information theoretic feature clustering algorithm [16] has been used to create compact, pyramid-based image representation for object and scene recognition, while a sparse coding-based, SPM kernel [17] was proposed to capture salient image properties for use in image categorization, by applying sparse coding and spatial max pooling to scale-invariant feature transform (SIFT) descriptors. Spatial pyramid image representation, achieved by computing histograms of SIFT descriptors [18] extracted from image sub-regions, has been proposed. In a separate study, several state-of-the-art visual descriptors [19] were evaluated, using classification accuracy ratings for four benchmark scene data sets, such as an eight-outdoor-scene data set, a 15-scene data set, a 67-indoor-scene data set, and the SUN397 data set. The major contribution of this study was the integration of proposed Ohta Color-GIST wavelet descriptors and CENTRIST (spatial pyramid representation) descriptors, to recognize complex indoor versus outdoor scenes for MAV navigation in GPS-denied indoor and outdoor environments. Finally, several additional scene recognition methods [20–22] have been proposed, for indoor versus outdoor scene and indoor scene categorization.

## Related scene recognition visual descriptors

Visual descriptors—such as SIFT-ScSPM, SIFT-LLC, SIFT-SPM, histogram of oriented gradients (HOG)-SPM, Enhanced-GIST, CENTRIST, CENTRIST (spatial pyramid representation), speeded-up Robust Features (SURF)-SPM, Color-GIST descriptors, Ohta Color-GIST wavelet descriptors, and spatial color gist wavelet descriptors (proposed visual descriptors)—have been used for indoor versus outdoor scene categorization. Several of the feature extraction methods used in these descriptors for scene categorization have been discussed in the following sub-sections.

### Sift

**Fig. 2:**
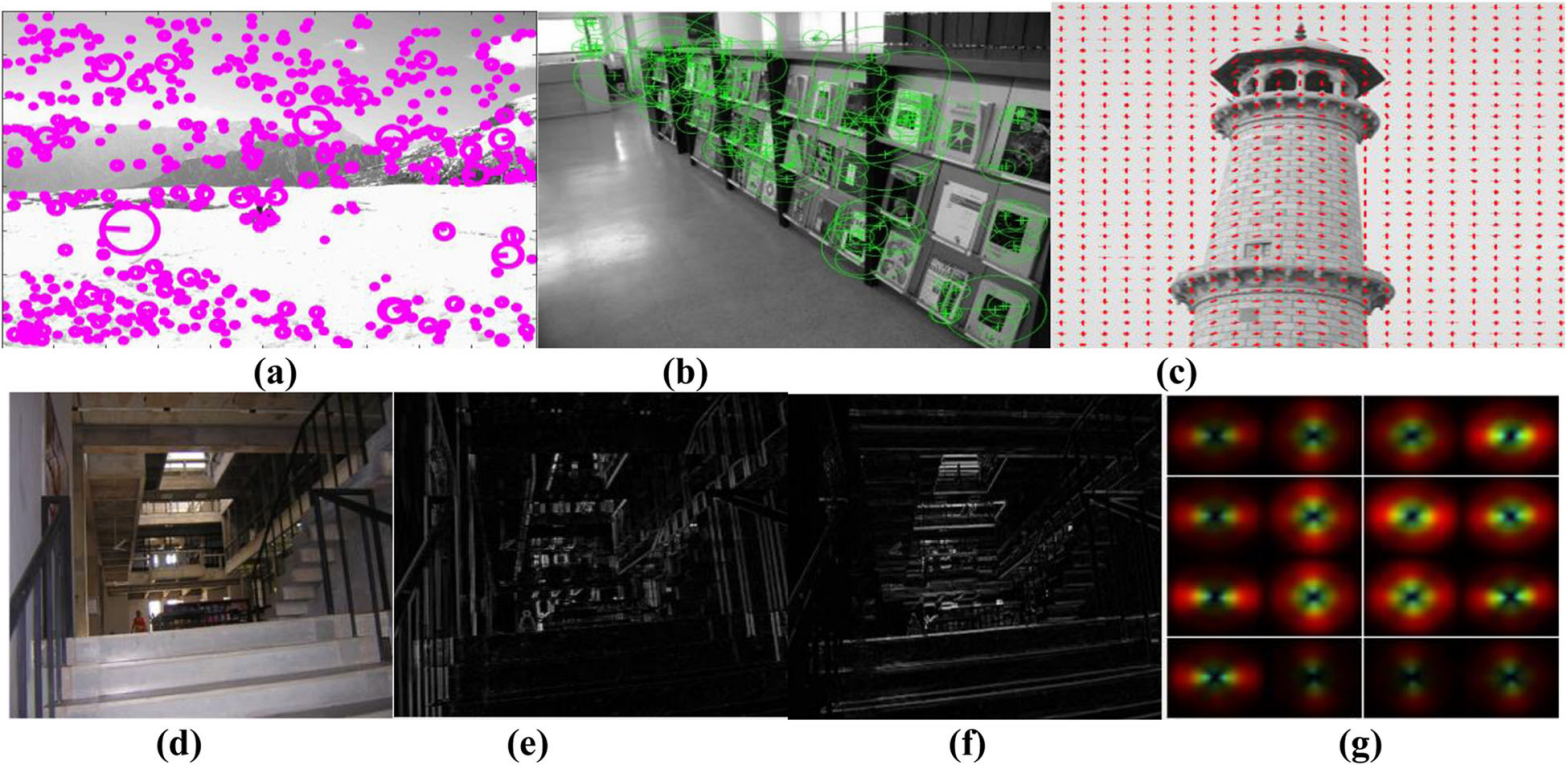
Illustration of visual descriptors. **a** Scale-invariant feature transform key points detected in an outdoor image; **b** Speeded up robust features key points detected in an indoor image; **c** Histogram of oriented gradients features detected in an outdoor image; **d** Input indoor image; **e** Horizontal directional morphological gradient; **f** Vertical directional morphological gradient; **g** GIST descriptor

SIFT keypoints extracted using the SIFT algorithm proposed by Lowe [23] are immune to rotation, translation, and image illumination and scaling changes. In this method, scale-space extreme detection, keypoint localization, orientation assignment, and calculation of keypoint descriptors are the four steps involved in SIFT descriptor extraction. Difference Gaussian filters are applied to the image frames to identify stable keypoint locations in scale space, in a step where, to detect stable keypoints, unstable keypoints below the threshold value are discarded. For each stable keypoint, orientations are assigned by computing the local image gradient, before gradient magnitudes and orientations are computed for each keypoint, to extract any SIFT descriptor in the 16 × 16 neighborhood of the pixel. Gradient magnitude and orientation are weighted using a Gaussian window around the location of the keypoint, to create orientation histograms with 8 bins over 4 × 4 (16 regions) sub regions, so that a 128-element, feature vector SIFT descriptor is extracted from each 4 × 4 sub-region. SIFT key points extracted from an indoor image are shown in Fig. 2a, in which the circle center and radius represent the detected SIFT keypoints and the average keypoint scale, respectively. Average keypoint orientation is represented by the arrow inside the circle.

### Surf

As for the SIFT descriptor, a scale invariant and rotation invariant visual descriptor [24] has been proposed, using determination of a hessian matrix. In this method, keypoints are selected from the detected SURF interest points in multi-scale space, using non-maximum suppression. Next, within a sliding orientation window, the sum of all Haar wavelet responses is calculated, to estimate the orientation of the keypoint with the longest vector. The Haar wavelet responses for each 4 × 4 sub-region around the key point are computed, in the horizontal (*d*_*x*_) and the vertical (*d*_*y*_) directions, so that, for each 4 × 4 sub-region, the feature vector is denoted as *v* = (∑*d*_*x*_, ∑*d*_*y*_,  ∑ | *d*_*x*_| ,  ∑ | *d*_*y*_| ). SURF key points are extracted as shown in Fig. 2b. The circle center and radius represent the detected SURF keypoints and the average keypoint scales, respectively. Average keypoint orientation is represented by the arrow inside the circle.

### Hog

The HOG descriptor [25] was proposed to detect pedestrians in grayscale images. In the HOG method, local gradient orientation histograms are computed from grayscale images, to extract the HOG descriptor. Firstly, image gradients along the horizontal and vertical directions are computed, followed by dividing the image into circular or rectangular connected regions—each with the dimensions of 16 × 16 pixels—called cells. Thirdly, gradient orientation histograms of are computed within each cell’s pixels, and each cell’s pixels contribute to the weighted score for a histogram. Finally, the L2-norm method is used to normalize cell histograms (9 bins) to obtain the HOG descriptor. A HOG descriptor detected in a grayscale image for 16 × 16 pixel cell size can be seen in Fig. 2c.

### Centrist

**Fig. 3:**
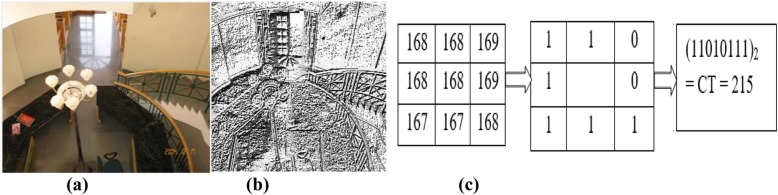
Census transformed output. **a** Indoor image; **b** Census transformed Indoor image; **c** Census transformed value

CENTRIST is a scene categorization visual descriptor developed [26] by replacing each pixel intensity value in a grayscale image with a census transform (CT) value. In this method, to convert the input grayscale image into a Census Transformed image (Fig. 3b) the center pixel intensity is compared with those of its 3 × 3-pixel neighborhood. Bit “1” is assigned to the neighboring pixel if the center pixel value is greater than the value of the neighboring pixel; otherwise the pixel value is set to “0”. After obtaining the CT value (Fig. 3c), histograms of CT values are used to obtain the 256-dimensional CENTRIST descriptor (not using principal component analysis). To extract CENTRIST descriptor spatial representation, an indoor or outdoor image is divided into 31 blocks (25 + 5 + 1), for a level 2, 1, and 0 split in a spatial pyramid. The CENTRIST descriptors extracted from the 31 image blocks are then concatenated, to produce a spatial representation of the 7936 (31 × 256) dimensional CENTRIST descriptor. Therefore, to reiterate, CENTRIST descriptors with spatial pyramid representation and CENTRIST descriptors differ from each other in the feature extraction stage.

### Enhanced-GIST descriptor

The Enhanced-GIST descriptor method was proposed [27] to recognize corridors, staircases, and room types, in indoor scenes, by encoding the spatial envelope and geometric structure of the indoor scene. In this method, a bank of 32 Gabor filters is applied to an indoor and outdoor grayscale image (256 × 256 pixels), at 4 scales and 8 orientations, to produce 32 feature maps. These 32 feature maps are then divided into 4 × 4 grids, and the filtered outputs within each of the 16 regions are averaged, to produce a 512-dimensional (16 × 32) GIST descriptor (Fig. 2g). Directional morphological gradient [28] features (Fig. 2e and f) are extracted for horizontal and vertical direction α, by using a line structuring element, as shown in Eq. (1):

1 $$ g{L}_{\alpha }(f)=\delta {L}_{\alpha }(f)-\varepsilon {L}_{\alpha }(f) $$

where *δL*_*α*_(*f*) denotes the dilated image and *εL*_*α*_(*f*) denotes the eroded image. Histograms of horizontal and vertical directional morphological gradient (HODMG) features are used for scene classification. GIST (512-dimensional) and HODMG (512-dimensional) descriptors are then combined, to produce 1024-dimensional Enhanced-GIST descriptors.

### Bag-of-words algorithms

Bag-of-words algorithms—such as SIFT-SPM, SIFT- ScSPM, and SIFT-LLC—have been employed for indoor versus outdoor scene classification. In the feature extraction phase, dense SIFT features are extracted from a regular grid, and quantized into discrete visual words, and the SPM algorithm was proposed [18] to categorize natural scenes. To extract SIFT-SPM, HOG-SPM, and SURF-SPM from indoor and outdoor images, the input image is first divided into regular grids (grid spacing of 8 pixels), and SIFT, SURF and HOG descriptors are extracted from each grid. The visual vocabulary is then formed, by applying k-means clustering to the extracted features. The vector quantization (VQ) technique is then applied, to form discrete visual words, with local features represented based on the trained dictionary (codebook). Spatial histograms of coded features constitute the final feature vectors. In the sparse coding-based, SPM method proposed by Yang et al. [17], the VQ method was replaced with the sparse coding method, to quantize the local features used in SPM. In SPM and ScSPM, the histograms and the max operator are used for spatial pooling, and locality-constrained linear coding (LLC) [29] is used for image classification. Linear-weighted combinations of similar bases are learned from the trained visual words, and represent local features.

### Wavelet descriptors

**Fig. 4:**
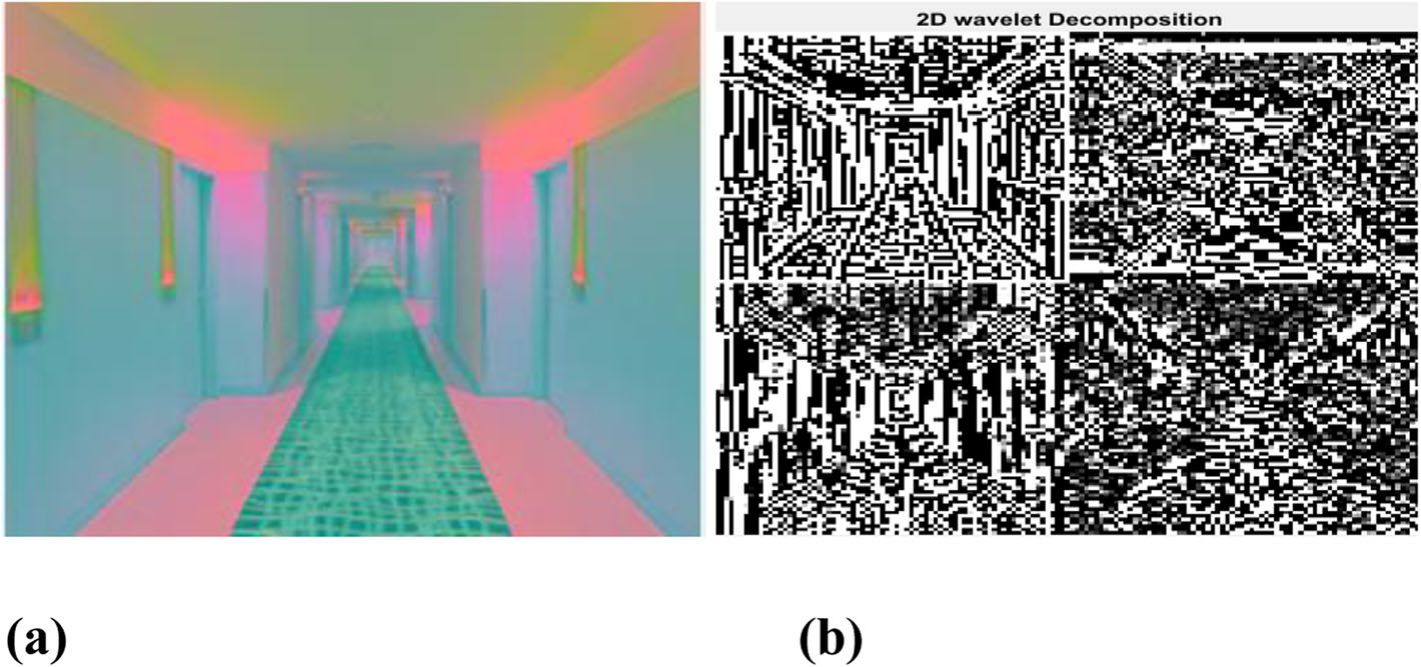
Wavelet descriptors. **a** CIELAB color space image; **b** 2 dimensional wavelet decomposition output

In this method, an input RGB image is converted into CIELAB (Lab) color space, and the resultant Lab color space image is re-sized with a scale factor value of 1/16. Wavelet descriptors [30] are computed by means of three-level wavelet decomposition, using a Biorthogonal wavelet transform. The Lab color space and 2-D wavelet decomposition image are shown in Fig. 4. The 2-D (2 dimensional) wavelet decomposition is performed on the “L” channel of the Lab color space, and the approximation coefficients at level 3 are extracted using biorthogonal wavelets (Bior 2.6).

Laplacian filtering is then applied to the extracted approximation coefficients, to obtain the Laplacian-filtered coefficients. Finally, the percentage of wavelet energy for 2-D wavelet decomposition—corresponding to the approximation coefficients (Laplacian-filtered coefficients), and the horizontal, vertical, and diagonal detail coefficients—is computed, and is used as a feature vector for indoor and outdoor image classification.

### Color-GIST descriptors

**Fig. 5:**
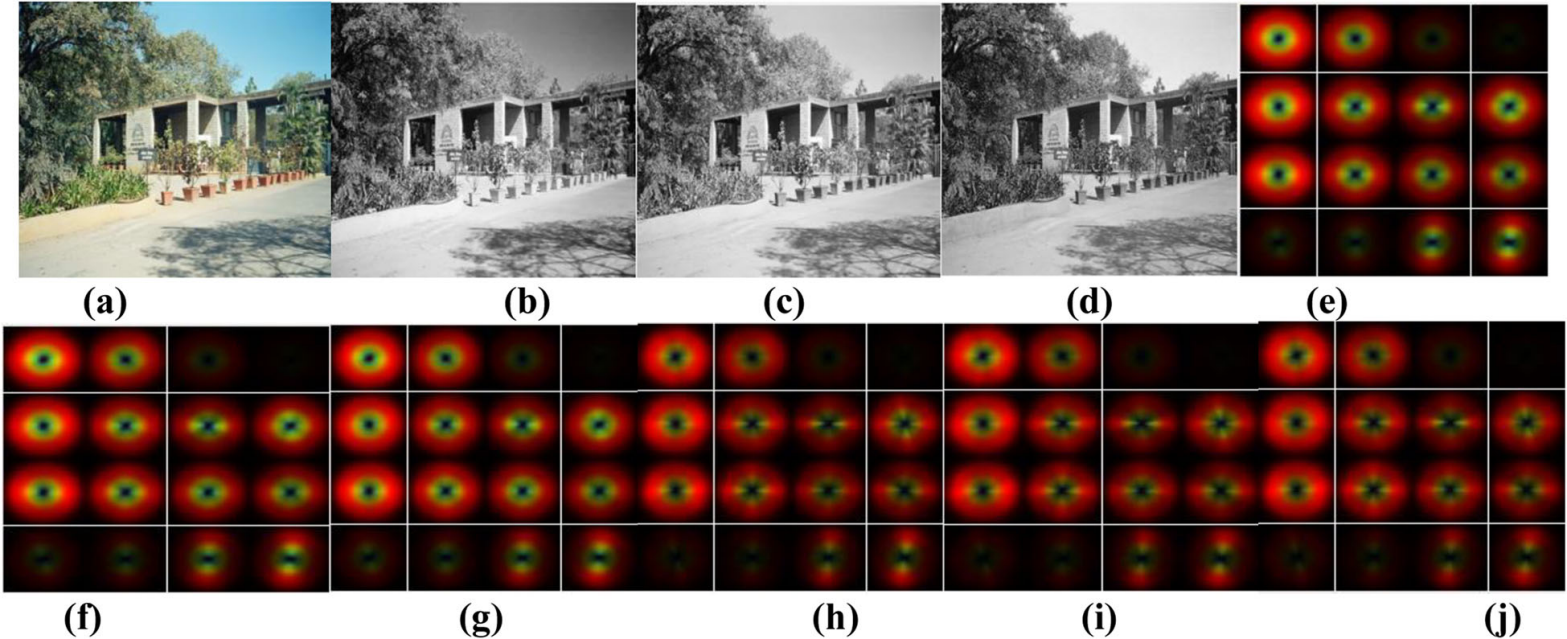
Visual illustration of Color-GIST descriptor.**a** RGB outdoor image; **b** Red channel (RGB image); **c** Green channel (RGB image); **d** Blue channel (RGB image); **e**-**g** Color-GIST descriptor extracted applying 32 Gabor filters at 4 scales and 8 orientations from red channel (**e**) green channel (**f**) and blue channel (**g**); **h**-**j** Color-GIST descriptor extracted applying 64 Gabor filters at 4 scales and 16 orientations from red channel (**h**) green channel (**i**) and blue channel (**j**)

Visual perception of a scene from red, green, and blue channel, RGB indoor and outdoor images, at a glance, is known as Color-GIST recognition of an indoor versus outdoor scene. Red channel, blue channel and green channel information is extracted from the input RGB image (resolution: 256 × 256 pixels). For the red channel, blue channel, and green channel, GIST descriptors [31] are extracted by applying 64 Gabor filters, at 4 scales and 16 orientations, to produce 64 feature maps. In each channel, the resultant maps are divided into 16 regions, and the filtered outputs within each region are then averaged to produce 1024-dimensional (64 × 16) descriptors. Color-GIST descriptors (Fig. 5) are extracted by concatenating the GIST descriptors extracted from each channel, to produce a 3072-dimensional (1024 × 3) descriptor.

## Methods

**Fig. 6:**
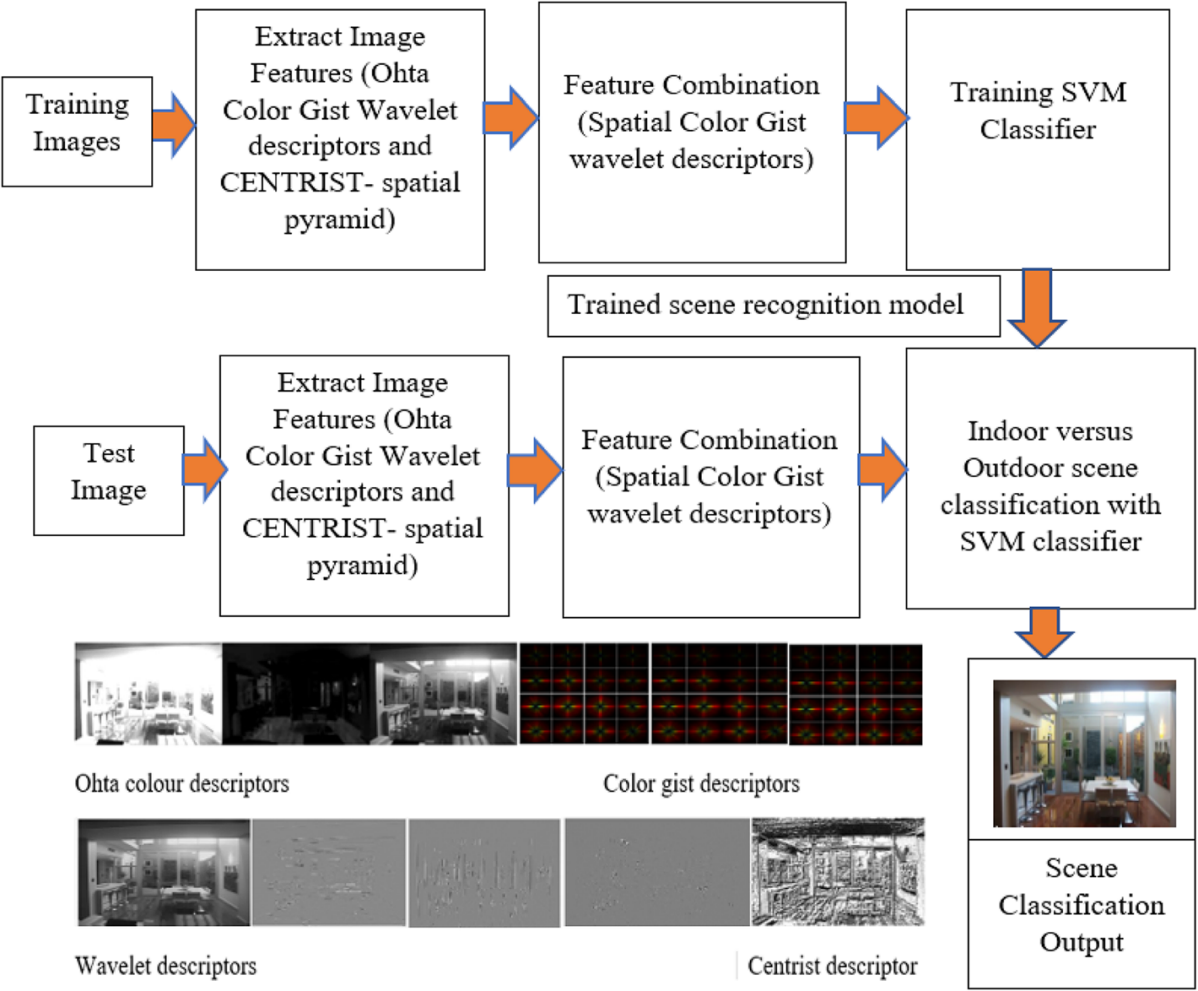
The block diagram of the proposed method

Real time indoor and outdoor scene recognition capabilities are needed, in order to navigate an MAV successfully. By recognizing scenes as indoor or outdoor, an MAV can follow a navigation strategy suitable for the performance of high level missions. A block diagram for a proposed indoor versus outdoor scene recognition framework is shown in Fig. 6.

The indoor versus outdoor scene recognition model consists of training and testing stages. In the training stage, visual descriptors, such as Ohta Color-GIST wavelet descriptors and CENTRIST (spatial pyramid) descriptors, are extracted from indoor and outdoor training images. The extracted SCGWDs are learned using a support vector machine (SVM) classifier, with linear and nonlinear kernels. In the testing stage, SCGWDs are extracted from the test image, and the SVM classifier with linear and nonlinear kernel classify the scene category as indoor or outdoor based on the scene recognition model learned at the training stage.

This study included comparative testing between several state-of-the-art visual descriptors and the proposed SCGWD, from both experimental and methodological perspectives, for indoor versus outdoor scene categorization tasks performed in the challenging Indian Institute of Technology Madras – Scene Classification Image Database two (IITM-SCID2), an Indoor-Outdoor Dataset, and the Massachusetts Institute of Technology indoor scene classification dataset ((MIT)-67). In our study, several existing, state-of-the art visual descriptors have been applied to the challenge of indoor versus outdoor scene categorization. The proposed SCGWD, Ohta Color-GIST wavelet descriptors, Ohta Color-GIST descriptor, and Enhanced Ohta color histogram descriptors have been compared with state-of-the-art visual descriptors, including CENTRIST-spatial pyramid, Color-GIST descriptors, Wavelet descriptors, Census Transform Histogram (CENTRIST), SIFT transformed with Sparse coding-based SPM (SIFT-ScSPM), SIFT with Locality-Constrained Linear Coding (SIFT-LLC), SIFT with SPM (SIFT-SPM), HOG with SPM (HOG-SPM), Speeded Up Robust Features with SPM (SURF-SPM), and Enhanced-GIST descriptors. The major contribution from the study has been integration of the proposed Ohta Color-GIST wavelet descriptors with CENTRIST (spatial pyramid) descriptors, to categorize indoor versus outdoor scene images for MAV navigation in GPS-denied indoor and outdoor environments.

### Enhanced Ohta color histogram descriptors (proposed visual descriptors)

Ohta color space [32] is a linear transformation of RGB color space, in which color channels are computed for the indoor and outdoor image as follows:

2 $$ {\displaystyle \begin{array}{l}\mathrm{I}1=\mathrm{R}+\mathrm{G}+\mathrm{B}\\ {}\mathrm{I}2=\mathrm{R}-\mathrm{B}\\ {}\mathrm{I}3=\mathrm{R}-2\mathrm{G}+\mathrm{B}\end{array}} $$

For each color channel, histogram features are extracted, using 32, 64, 128, and 256 bins, respectively, and then these features are concatenated, to form the Enhanced Ohta color histogram descriptors (480 dimensional descriptor).

### Ohta color-GIST descriptors and Ohta color-GIST wavelet descriptors (proposed visual descriptors)

Ohta color histogram descriptors and Color-GIST descriptors are combined to produce Ohta Color-GIST descriptors (3552-dimensional descriptor (3072 + 480)). For each indoor and outdoor image, the Enhanced Ohta color histogram descriptors, Color-GIST descriptors, and wavelet descriptors are combined, to produce the proposed Ohta Color-GIST wavelet descriptors (480 + 3072 + 22 = 3574-dimensional descriptor).

### SCGWDs (proposed visual descriptor)

SCGWDs are a new visual descriptor, created for the indoor versus outdoor scene classification task by combining Ohta Color-GIST wavelet descriptors with CENTRIST (spatial pyramid representation) descriptors. In this method, Enhanced Ohta color histogram descriptors were initially extracted, using 32, 64, 128, and 256 histogram bins. Then, Color-GIST descriptors were extracted from the image frame red, green, and blue channels, by applying 64 Gabor filters, at four scales and sixteen orientations, to obtain 64 feature maps. We then divided the 64 feature maps into 4 × 4 grids and averaged the Gabor-filtered outputs to obtain the Color-GIST descriptors. In the next stage, wavelet descriptors were extracted by computing 2-D wavelet decomposition at level 3 on the “L” channel of the Lab color space image. The energy coefficients from the Laplacian-filtered approximation coefficients, and the horizontal, vertical, and diagonal detail coefficients were then combined, to produce wavelet descriptors. After this, CENTRIST (spatial pyramid) descriptors were extracted by dividing the image into 31 blocks and concatenating the histogram of the Census Transformed values into 31 image blocks, to produce spatial pyramid representations of the CENTRIST (spatial pyramid) descriptors. Finally, Enhanced Ohta color histogram descriptors, Color-GIST descriptors, wavelet descriptors, and CENTRIST (spatial pyramid) descriptors were combined, to produce the proposed SCGWDs.

## Implementation of indoor versus outdoor scene visual descriptors

In this section, we have discussed using state-of-the-art visual descriptors and the proposed Ohta color-GIST descriptors, Ohta color-GIST wavelet descriptors, and SCGWDs for indoor versus outdoor scene classification.

To extract the Enhanced-GIST descriptor, the input grayscale image was divided into 16 regions, and 32 Gabor filters were applied, at 4 scales and 8 orientations, to obtain 32 GIST features. The 32 Gabor-filtered output responses within 16 regions were then averaged, to produce a 512-dimensional (16 × 32) GIST descriptor. Finally, the extracted GIST descriptors (512-dimensional) were combined with HODMG features (also 512-dimensional), to produce a 1024-dimensional, Enhanced-GIST descriptor.

Three different feature vector encoding methods—SPM, LLC and ScSPM—were used to encode the SIFT-based descriptor. To implement the SIFT-ScSPM, SIFT-LLC, and SIFT-SPM descriptors, dense SIFT features were extracted from overlapping 16 × 16 patches, on dense grids, established every 8 pixels.

SIFT-ScSPM descriptors were extracted by applying sparse coding to the dense SIFT features, and then a linear SPM kernel, based on this sparse coding, was obtained by applying spatial max pooling. For SIFT-ScSPM use in our study, the sparse constraint parameter value was set at 0.15, and a trained dictionary of 1024 words was used. This 1024-word, feature dictionary was obtained by applying the K-means clustering algorithm to local features. Finally, using the ScSPM algorithm, global features were produced from the dense SIFT descriptors. The feature vector encoding capabilities of the SIFT-ScSPM, SIFT-LLC, and SIFT-SPM descriptors were evaluated and compared—with the results shown in Table 1.

**Table 1:** Scene categorization performance on the Indian institute of technology madras-scene classification image database two dataset

No	Algorithms	CR	Precision	Recall	F-measure	AUC
1	CENTRIST-spatial pyramid (RBF)	92.93%	92.93%	92.91%	92.92%	92.92%
2	CENTRIST-spatial pyramid (linear)	92.53%	92.53%	92.54%	92.53%	92.54%
3	Color-GIST descriptors (RBF)	89.59%	89.58%	89.58%	89.58%	89.37%
4	Color-GIST descriptors (linear)	81.73%	81.77%	81.68%	81.73%	81.68%
5	Wavelet descriptors (RBF)	75.05%	75.10%	75.09%	75.09%	75.09%
6	CENTRIST (linear)	85.06%	85.08%	85.09%	85.08%	85.10%
7	CENTRIST (RBF)	86.24%	86.27%	86.28%	86.28%	86.28%
8	SIFT-ScSPM (linear)	92.92%	92.92%	92.94%	92.93%	92.94%
9	SIFT-LLC (linear)	92.53%	92.52%	92.54%	92.53%	92.55%
10	SIFT-SPM (Chi-square)	86.05%	87.42%	85.84%	86.62%	85.84%
11	HOG-SPM (Chi-square)	80.74%	86.31%	80.32%	83.20%	80.32%
12	SURF- SPM (Chi-square)	84.47%	88.34%	84.13%	86.19%	84.14%
13	Enhanced-GIST (RBF)	89.98%	90.19%	89.90%	90.05%	90%
14	Enhanced-GIST (linear)	84.28%	84.40%	84.21%	84.21%	84.22%
15	Enhanced Ohta color histogram descriptors (RBF)-proposed	76.03%	77.04%	76.23%	76.63%	76.23%
16	Ohta Color-GIST descriptors (RBF)-proposed	90.57%	90.56%	90.56%	90.56%	90.57%
17	Ohta Color-GIST descriptors (linear)-proposed	88.21%	88.21%	88.21%	88.21%	88.21%
18	Ohta Color-GIST wavelet descriptors (linear)-proposed	88.61%	88.61%	88.59%	88.60%	88.59%
19	Ohta Color-GIST wavelet descriptors (RBF)-proposed	90.57%	90.56%	90.56%	90.56%	90.57%
20	spatial color-gist wavelet descriptors (linear) -proposed	95.29%	95.28%	95.28%	95.28%	95.28%
21	spatial color-gist wavelet descriptors (RBF)-proposed	95.48%	95.50%	95.47%	95.48%	95.47%

*AUC* Area under the receiver operating characteristic curve, *CR* Classification rate, *RBF* Radial basis function kernel, *SIFT-LLC* SIFT with locality-constrained linear coding, *SIFT-ScSPM* SIFT with sparse coding based spatial pyramid matching, *SIFT-SPM* SIFT with spatial pyramid matching, *SPM* Spatial pyramid matching, *HOG* Histogram of oriented gradients, *SURF* Speeded up robust features, *CENTRIST* Census transform histogram

SIFT-SPM descriptors were extracted by applying the SPM algorithm from dense SIFT descriptors to global descriptors from the indoor and outdoor image. Three pyramid layers in the SPM scheme, and a codebook size of 400, were used here to extract the SIFT-SPM descriptors. A visual vocabulary of 400 visual words was obtained from the training set, by applying K-means clustering to a random subset of patches, and then local features were quantized, using the trained visual words. Finally, SIFT-SPM descriptors with 8400 bins were obtained, by concatenating the quantized SIFT feature histograms obtained from the indoor and outdoor image.

SIFT-LLC descriptors were extracted from the image by applying the LLC algorithm to dense SIFT features. A 1024-entry codebook, trained using the K-means clustering technique, was used to obtain the LLC codes from indoor and outdoor images. In the LLC feature extraction stage in our study, the K-nearest neighbor value was set to 5. Finally, max pooling was applied to the LLC codes of the dense SIFT descriptors, to produce SIFT-LLC descriptors.

HOG-SPM descriptors were extracted from overlapping 16 × 16 patches, by extracting local features from a dense grid which had a step size of 8 pixels. Firstly, image gradients were computed along the horizontal and vertical directions of the grayscale indoor and outdoor images, and then histograms were computed for multiple orientations, to produce HOG features. Global features were obtained by applying the SPM algorithm to the HOG descriptors. In HOG-SPM for this study, 3 pyramid layers (4200 dimension) and a 200-word codebook were used.

SURF-SPM descriptors were extracted by detecting SURF interest points (100 interest points) in 256 × 256 pixel resolution, grayscale images. Global features (SURF-SPM) were obtained using the SPM algorithm, and in SURF-SPM, as used here, 3 pyramid layers (8400-dimension) and a codebook size of 400 and were used.

CENTRIST descriptors were extracted by comparing the center pixel to the intensity values of its 3 × 3 pixel neighborhood. Bit “0” was assigned to the neighboring pixel if the center pixel value was less than the neighboring pixels, otherwise bit “1” was assigned. The CENTRIST descriptors extracted from indoor and outdoor images were 256-dimensional descriptors. Spatial representation of CENTRIST descriptors was extracted by dividing the input image into 31 blocks, and the obtained histogram of Census Transformed values were concatenated into 31 blocks, to produce a 7936-dimensional descriptor.

To extract Wavelet descriptors, RGB input images were converted into CIELAB color space images. 2-D Wavelet decomposition, using biorthogonal wavelets (Bior 2.6), was applied, on the “L” channel of the Lab color space image. Wavelet energy coefficients—such as approximation coefficients (Laplacian-filtered coefficients), and horizontal, vertical, and diagonal detail coefficients—were extracted at level 3 and used as feature vectors. To extract the Enhanced Ohta color histogram descriptors, RGB images were converted into Ohta color space channels I1, I2, and I3, and Ohta color channel histograms were computed with 32, 64, 128, and 256 bins. The color histogram features were then concatenated, to produce Enhanced Ohta color histogram descriptors.

To extract Color-GIST descriptors, input RGB image red, green, and blue channels were each divided into 16 regions, and 64 Gabor filters were applied, at 4 scales and 16 orientations, to obtain 64 GIST feature maps. The 64 Gabor-filtered output responses within 16 regions were then averaged, to produce a 1024-dimensional (16 × 64) GIST descriptor. GIST descriptors extracted from each channel were then combined, to produce 3072-dimensional (1024 × 3) Color-GIST descriptors.

The extracted Enhanced Ohta color histogram descriptors and Color-GIST descriptors were combined, to produce the proposed Ohta Color-GIST descriptors—which were 3552-dimensional descriptors. The Enhanced Ohta color histogram descriptors, the Color-GIST descriptors, and the wavelet descriptors were then combined, to produce the proposed Ohta Color-GIST wavelet descriptors (3574-dimensional descriptors).

SCGWDs were then extracted for indoor and outdoor images by combining the Enhanced Ohta color histogram descriptors, the Color-GIST descriptors, the wavelet descriptors, and the CENTRIST (spatial pyramid) descriptors.

## Experimental evaluation and results

This section presents an experimental evaluation of the proposed visual descriptor’s performance, with respect to current, state-of the-art visual descriptors. All visual descriptors were evaluated using the IITM-SCID2 Dataset and Indoor-Outdoor Dataset, using five performance measures—CR, precision, recall, F-measure, and area under the curve (AUC).

### Dataset for indoor versus outdoor scene recognition

Scene classification experiments were conducted using the IITM-SCID2 and Indoor-Outdoor datasets. We used complex and confusing indoor and outdoor images, which required visual descriptors with strong discriminative ability to classify the indoor and outdoor scenes. Indoor scene classification experiments were conducted on the MIT-67 Dataset.

The IITM-SCID2 Dataset is a challenging benchmark dataset containing 902 images of indoor and outdoor scenes. From this dataset, 193 indoor images and 200 outdoor images were used in the training phase, to train the SVM classifier, while in the testing phase, 249 indoor images and 260 outdoor images were used, based on the trained scene recognition model. The Indoor-Outdoor Dataset consists of eight outdoor scene categories, including tall buildings, cityscapes, streets, highways, mountains, forests, open country, and coastal images, in a 4485-image dataset, and three indoor scene categories, including corridors, staircases, and rooms. Real time image frames were acquired from MAVs, with an image resolution of 1280 × 720 pixels. The dataset contained 1100 training images (300 indoor and 800 outdoor images), and 550 testing images (150 indoor and 400 outdoor images). The MIT-67 Dataset contains 15,620 images in 67 categories of complex indoor scenes. Here, 100 images or so from each of the 67 categories were chosen, so that around 6700 images were used for training and testing the classifier, using ten-fold cross validation, respectively.

### Performance measures and kernels used for indoor versus outdoor scene recognition

In this paper, five different performance measures, including CR, precision, recall, f-measure, and AUC of the receiver operating characteristic (ROC) curve were used to assess indoor versus outdoor scene classification performance. In out testing, CR was defined as the percentage of correctly categorized test images, and four measures—true positives (TP), false positives (FP), false negatives (FN), and true negatives (TN)— could be computed, using the obtained confusion matrix.

Precision rate was expressed as shown in Eq. (3):

3 $$ P=\frac{TP}{TP+ FN} $$

where *TP* and *FN *represent the TP and FN, respectively.

Recall rate was expressed as shown in Eq. (4):

4 $$ R=\frac{TP}{TP+ FP} $$

where *TP *and* FP *represent the TP and FP, respectively.

The F-measure was defined as shown in Eq. (5):

5 $$ F=\frac{2 PR}{P+R} $$

where* P* and *R* represent the precision rate and recall rate, respectively.

Area under the ROC curve, or AUC, was used as a performance measure to evaluate the indoor versus outdoor scene classification algorithm.

In this work, SVM with three different types of kernels—linear, RBF, and chi-squared—were used to classify the indoor and outdoor scenes.

The linear kernel for SVM classification could be computed as shown in Eq. (6):

6 $$ K\left({x}_i,{x}_j\right)={x}_i.{x}_j $$

where *x*_*i*_ and *x*_*j*_ represent two feature vectors.

The RBF kernel for SVM classification could then be computed as shown in Eq. (7):

7 $$ K\left({x}_i,{x}_j\right)=\mathit{\exp}\left\{-\gamma {\left\Vert {x}_i-{x}_j\right\Vert}^2\right\} $$

where γ is a positive scalar, and *x*_*i*_ and *x*_*j*_ denote two feature vectors.

The Chi-square kernel could be then computed as shown in Eq. (8):

8 $$ k\left(x,y\right)=1-\sum \limits_{i=1}^N\frac{2{\left({x}_i-{y}_i\right)}^2}{\left({x}_i+{y}_i\right)} $$

where *x*_*i*_ and *y*_*i*_ represent two feature vectors. The Recognition rate, Precision (P), Recall (R), F-measure (F) and AUC were calculated, and the performance of the visual descriptors with SVM classifiers was evaluated using the IITM-SCID2 and Indoor-Outdoor datasets.

### Classification results using the IITM-SCID2, indoor-outdoor, and MIT-67 datasets

Indoor versus outdoor scene classification results achieved on the IITM-SCID2 Dataset and Indoor-Outdoor Dataset using state-of-the-art visual descriptors and three proposed visual descriptors—Ohta color-GIST descriptors, Ohta color-GIST wavelet descriptors and SCGWD—have been listed in Tables 1 and 2. Scene classification experiments were conducted on a laptop computer equipped with an Intel i7-7500U CPU, operating at 2.70 GHz, and using 16 GB of RAM.

**Table 2:** Scene categorization performance on the Indoor Outdoor dataset

No	Algorithms	CR	Precision	Recall	F-measure	AUC
1	CENTRIST-spatial pyramid (RBF)	97.27%	97.47%	95.62%	96.54%	95.63%
2	CENTRIST-spatial pyramid (linear)	95.81%	95.36%	94%	94.67%	94%
3	Color-GIST descriptors (RBF)	89.63%	92.89%	81.41%	86.77%	81.42%
4	Color-GIST descriptors (linear)	83.27%	78.84%	81.83%	80.31%	81.83%
5	Wavelet descriptors (RBF)	74.54%	69.11%	71.45%	70.26%	71.46%
6	CENTRIST (linear)	93.63%	92.88%	90.83%	91.84%	90.83%
7	CENTRIST (RBF)	97.27%	96.14%	97.08%	96.61%	97.08%
8	SIFT-ScSPM (linear)	98.72%	98.49%	98.29%	98.39%	98.29%
9	SIFT-LLC (linear)	99.27%	98.88%	99.29%	99.08%	99.29%
10	SIFT-SPM (Chi-square)	57.81%	51.75%	52.04%	51.89%	52.04%
11	HOG-SPM (Chi-square)	71.81%	51.79%	50.20%	50.98%	50.21%
12	SURF- SPM (Chi-square)	73.27%	86.56%	51%	64.18%	51.00%
13	Enhanced-GIST (RBF)	97.81%	96.73%	97.87%	97.30%	97.88%
14	Enhanced-GIST (linear)	96.72%	94.98%	97.12%	96.04%	97.12%
15	Enhanced Ohta color histogram descriptors (RBF)-proposed	73.45%	65.11%	61.33%	63.16%	61.33%
16	Ohta Color-GIST descriptors (RBF)-proposed	98.73%	98.29%	98.50%	98.39%	98.50%
17	Ohta Color-GIST descriptors (linear)-proposed	96.72%	94.86%	97.33%	96.08%	97.33%
18	Ohta Color-GIST wavelet descriptors (linear)-proposed	97.27%	95.70%	97.70%	96.69%	97.71%
19	Ohta Color-GIST wavelet descriptors (RBF)-proposed	98.73%	98.29%	98.50%	98.39%	98.50%
20	Spatial Color-gist wavelet descriptors (linear) -proposed	99.45%	99.21%	99.42%	99.31%	99.42%
21	spatial color-gist wavelet descriptors (RBF)-proposed	99.82%	99.67%	99.87%	99.77%	99.88%

*AUC* Area under the receiver operating characteristic curve, *CR* Classification rate, *RBF* Radial basis function kernel, *SIFT-LLC* SIFT with locality-constrained linear coding, *SIFT-ScSPM* SIFT with sparse coding based spatial pyramid matching, *SIFT-SPM* SIFT with spatial pyramid matching, *SPM* Spatial pyramid matching, *HOG* Histogram of oriented gradients, *SURF* Speeded up robust features, *CENTRIST* Census transform histogram

Experimental results indicated that, among the two datasets (the benchmark IITM-SCID2 Dataset and the Indoor-Outdoor Dataset), the IITM-SCID2 Dataset was the most difficult and challenging dataset from which to classify indoor and outdoor images.

The benchmark, eight-outdoor-scene data set (Outdoor images) included in the Indoor-Outdoor Dataset was the easiest to classify, in comparison to the outdoor images in the IITM-SCID2 Dataset. The highest average CRs—95.48% and 99.82% using the RBF kernel, and 95.29% and 99.45% using the linear kernel—attained from the IITM-SCID2 Dataset and Indoor-Outdoor Dataset were achieved by the SCGWDs. CR, Precision, Recall, F-measure, and area under the ROC (AUC) performance measures indicated that the SCGWD achieved results better than those of the state-of-the-art visual descriptors.

The proposed method could be used as a scene classifier for navigating MAVs in indoor and outdoor environments. The results inferred that the combination of color features (Enhanced Ohta color histogram descriptors and color-GIST descriptors) and texture features [Wavelet descriptors and CENTRIST (spatial pyramid representation)] was very effective in classifying indoor and outdoor scenes.

Our study was a comparative study between several state-of-the-art visual descriptors and the proposed Ohta color-GIST descriptors, Ohta color-GIST wavelet descriptors and SCGWD, from both methodological and experimental perspectives, for indoor versus outdoor scene and indoor scene classification tasks. Indoor scene SCGWD classification results achieved using the MIT-67 dataset have been listed in Table 3, and CRs of 2.08% and 4.92% were obtained using the RBF and linear kernels.

**Table 3:** Computational cost calculation

Type of feature	SVM classifier method	IITM-SCID2 Dataset		Indoor-Outdoor Dataset		MIT-67 indoor scene classification Dataset	
		Recognition rate	Average time elapsed, in seconds per frame	Recognition rate	Average time elapsed, in seconds per frame	Recognition rate	Average time elapsed, in seconds per frame
Spatial color gist wavelet descriptors	linear kernel	95.29%	2.58	99.45%	2.80	4.92%	1.74
	RBF kernel	95.48%	2.60	99.82%	2.89	2.08%	2.12

*SVM* Support vector machine, *IITM-SCID2* Indian institute of technology madras-scene classification image database two, *RBF* Radial basis function kernel

These experimental results indicated that SCGWDs performed better for indoor versus outdoor scene categorization as opposed to indoor scene categorization. In fact, the SCGWDs were proposed mainly for indoor versus outdoor scene categorization, but descriptor effectiveness was also tested, and was found to be unsuitable for the indoor scene classification task, as the spatial layout, color, and texture information extracted from indoor scenes using the SCGWD was insufficient for recognition of indoor scenes.

Compared to other visual descriptors, SCGWD had the highest CR on the IITM-SCID2 Dataset and Indoor-Outdoor Dataset, as shown in Tables 1 and 2.

**Fig. 7:**
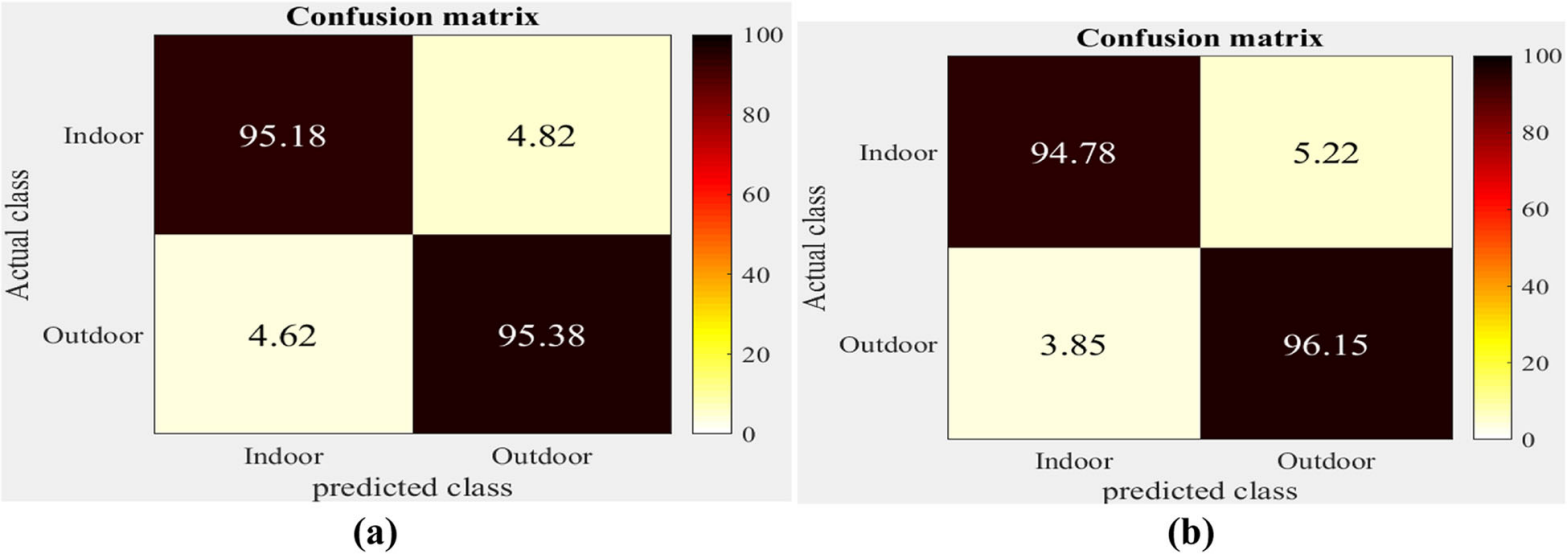
Confusion matrices obtained for spatial color gist wavelet descriptors on Indian institute of technology madras-scene classification image database two dataset. **a** Linear kernel; **b** Radial basis function kernel

**Fig. 8:**
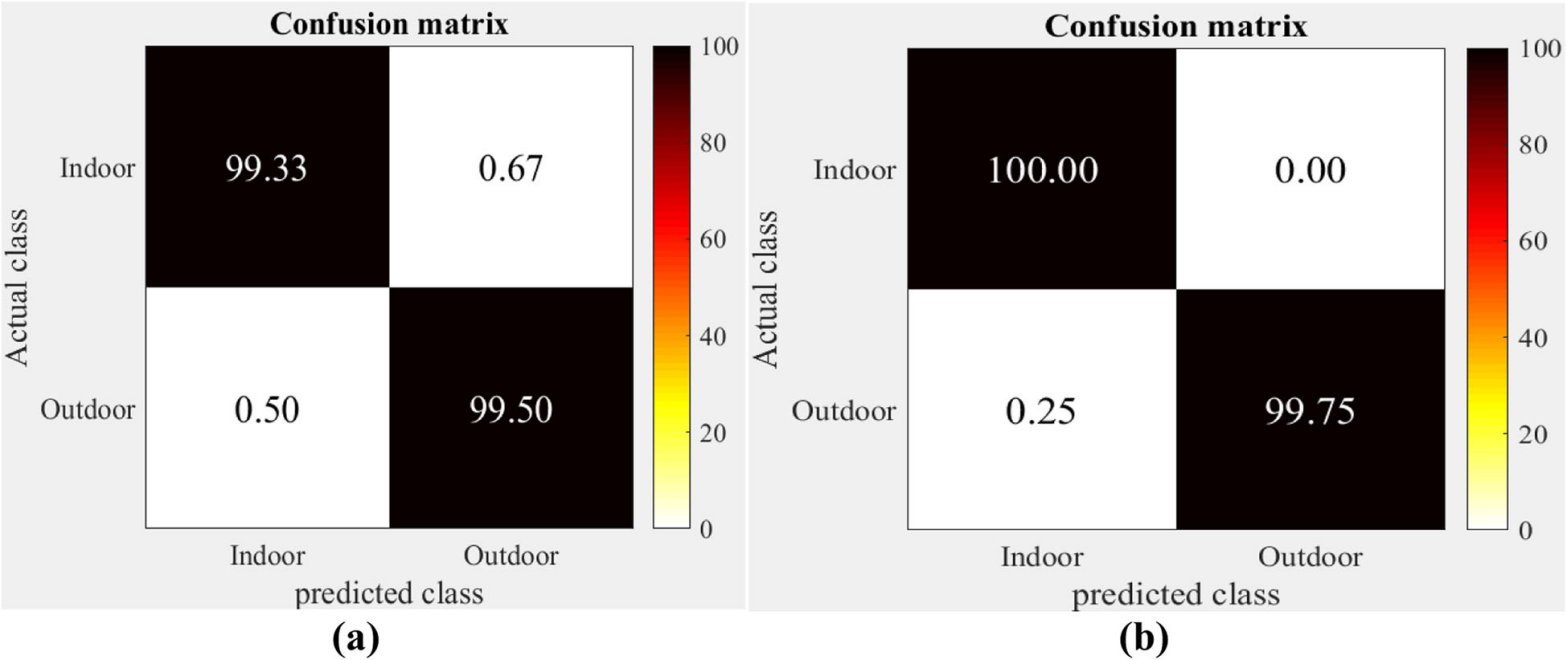
Confusion matrices obtained for Spatial color gist wavelet descriptors on Indoor Outdoor dataset. **a** Linear kernel; **b** Radial basis function kernel

The confusion matrices obtained when applying the SCGWD to the IITM-SCID2 and Indoor-Outdoor datasets are shown in Figs. 7 and 8, respectively. The rows and columns in the confusion matrices correspond to actual and predicted classes, respectively, while the diagonal values show the average CR for each indoor and outdoor image category.

Computation times taken, per frame, have been documented in Table 3.

## Conclusions

Indoor versus outdoor scene classification is difficult, due to intra-class variability, inter-class similarity in the case of indoor scenes, and the variability of outdoor scene content caused by different weather conditions and the nature of the objects involved in outdoor scenes. In this paper, a new visual descriptor, called the SCGWD and based on the combination of proposed Ohta color-GIST wavelet descriptors and CENTRIST (spatial pyramid representation) descriptors for Indoor-Outdoor scene classification task, has been described. The proposed new visual descriptor consists of color, texture, and spatial information content of the scene. When faced with the IITM-SCID2 dataset and Indoor-Outdoor Dataset, SCGWDs produced recognition rates of 95.48% and 99.82%, using SVM with an RBF kernel, and 95.29% and 99.45% using SVM with a linear kernel. Furthermore, using SCGWDs for scene classification, higher CR, precision, recall, and area under the ROC curve values were obtained, with respect to other, state-of-the-art visual descriptors.

In contrast, CRs of only 2.08% and 4.92% were obtained using RBF and linear kernels with the MIT-67 dataset, which showed that the SCGWD was unsuitable for use in the categorization of complex, 67 indoor scene image categories. Overall, however, we were able to conclude that the proposed scene recognition algorithm could be used as a scene classifier (Indoor versus Outdoor) for navigating MAVs.

## Data Availability

The datasets used and/or analyzed for indoor versus outdoor scene categorization and indoor scene categorization are available from the corresponding author on reasonable request.

## Abbreviations

2-D: 2 Dimensional
AR: Augmented reality
AUC: Area under the receiver operating characteristic curve
CENTRIST: Census transform histogram
CR: Classification rate
CT: Census transform
FN: False negatives
FP: False positives
GPS: Global positioning systems
HOG: Histogram of oriented gradients
HOG-SPM: HOG with spatial pyramid matching
IITM-SCID2: Indian institute of technology madras-scene classification image database two
LLC: Locality-constrained linear coding
MAV: Micro aerial vehicle
MIT: Massachusetts Institute of Technology
RBF: Radial basis function kernel
ROC: Receiver operating characteristic
ScSPM: Sparse coding based spatial pyramid matching
SIFT: Scale-invariant feature transform
SIFT-LLC: SIFT with locality-constrained linear coding
SIFT-ScSPM: SIFT with sparse coding based spatial pyramid matching
SIFT-SPM: SIFT with spatial pyramid matching
SPM: Spatial pyramid matching
SUN: Scene understanding
SURF: Speeded up robust features
SURF-SPM: SURF with spatial pyramid matching
SVM: Support vector machine
TN: True negatives
TP: True positives
VQ: Vector quantization
